# Systemic antibiotic treatment of cows with metritis early postpartum does not change the progression of uterine disease or the uterine microbiome at 1 month postpartum

**DOI:** 10.21203/rs.3.rs-4233045/v1

**Published:** 2024-04-12

**Authors:** Joao Gabriel Nascimento Moraes, Tamara B. Gull, Aaron C. Ericsson, Monica O. Caldeira, Tim J. Evans, Scott E. Poock, Matthew C. Lucy

**Affiliations:** Oklahoma State University; University of Missouri; University of Missouri; University of Missouri; University of Missouri; University of Missouri; University of Missouri

**Keywords:** Microbiome, uterus, antibiotics, ceftiofur

## Abstract

**Background:**

Postpartum uterine disease (metritis) is common in dairy cows. The disease develops within 1 week after calving and is associated with microbial dysbiosis, fever, and fetid uterine discharge. Cows with metritis have a greater likelihood of developing endometritis and infertility later postpartum. Antibiotic treatment is used to relieve symptoms of metritis but the capacity of antibiotic treatment to improve fertility later postpartum is inconsistent across published studies. We hypothesized that an antibiotic has only a short-term effect on the uterine microbiome and does not change the progression of disease from metritis to endometritis. To test this hypothesis, we studied the effects of systemic antibiotic given to cows diagnosed with metritis and healthy cows early postpartum on the development of endometritis and the uterine microbiome at 1 month postpartum.

**Results:**

Cows diagnosed with metritis were compared to healthy ones in a 2 × 2 factorial design, where they were either treated with an antibiotic (ceftiofur hydrochloride) at 7 to 10 days postpartum or left untreated. Cows were slaughtered at one month postpartum and the uterus was assessed for endometritis (presence of purulent material in the uterine lumen and inflammation in the endometrium) and uterine samples were collected for bacteriology and metagenomics (16S rRNA gene sequencing). As expected, the uterine microbiome at disease diagnosis had dysbiosis of typical metritis pathogens (e.g., Fusobacterium, Bacteroides, and Porphyromonas) in diseased compared with healthy cows. At one month postpartum, there was a tendency for more endometritis in metritis cows compared with healthy but antibiotic treatment had no effect on endometritis prevalence regardless of the original disease diagnosis. Likewise, when bacteria were cultured or sequenced, there were a greater number of species (culture) or amplicon sequence variants (ASV; sequencing) in the uterine lumen of cows with metritis. However, antibiotic treatment had no effect on the prevalence of cultured species or the composition of the detected ASV. The uterine microbiome at 1 month postpartum was associated with the clinical observation of the uterus (endometritis or healthy).

**Conclusions:**

Early postpartum antibiotic treatment only provides temporary resolution of uterine dysbiosis that is not sustained long-term. Failure to resolve the dysbiosis is associated with a greater prevalence of endometritis in cows with metritis, and the occurrence of endometritis significantly impacts fertility later postpartum.

## Introduction

Metritis (uterine infection after calving) can have a profound negative effect on the health and productivity of postpartum dairy cows causing fever, fetid uterine discharge, loss of appetite, and reduced milk production during early lactation [1–4]. Approximately one-third of postpartum dairy cows develop metritis within one week after calving [5–7] and nearly 50% of all deaths on farms occur within one month postpartum due to metritis or other complications that originate during calving [8]. Cows with metritis may fail to completely recover from the disease and develop chronic inflammation of the uterus (clinical or subclinical endometritis) that can last for several months [7, 9, 10]. If left untreated, endometritis places the cow at risk for failure to establish and maintain pregnancy when she is inseminated two to three months postpartum [1, 11–13]. The underlying mechanisms through which metritis can cause infertility or how the microbiome (both commensal and pathogenic organisms) interacts with normal processes of uterine tissue regeneration and repair in cows are not understood.

Metritis typically occurs within one week after calving and is associated with uterine microbial dysbiosis involving *Fusobacterium*, *Bacteroides*, and *Porphyromonas* [14]. These bacterial species damage the lining of the uterus and release lipopolysaccharide to cause the classical symptoms of metritis (fever, loss of appetite and fetid uterine discharge) [15]. Antibiotics are commonly used to treat metritis [16]. Ceftiofur is typically used because it does not pass into the milk [17]. Milk from cows treated with ceftiofur, therefore, can be sold without a withdrawal period. Ceftiofur leads to a rapid (within 2 d) shift in the uterine microbiome specifically targeting *Fusobacterium necrophorum* [18]. The decrease in the secretion of lipopolysaccharide from *Fusobacterium necrophorum* reduces the fever that, in turn, increases appetite and milk production [18]. Although ceftiofur will effectively relieve the symptoms of metritis [17, 19], the drug is not labeled for improving fertility postpartum when cows are inseminated (several months after cows are treated and the symptoms have subsided). Additional studies that followed the regulatory approval were poorly controlled with respect to testing the efficacy of ceftiofur for improving fertility [12, 20]. Antibiotic treatment failed to increase fertility of cows with metritis to a level found in healthy cows in two studies [21, 22] whereas a third study showed some improvement [23]. We hypothesize that the short-term changes in the microbiome following treatment demonstrated by Jeon et al. [18] are not sustained within the uterus (i.e., the pathogens reestablish dysbiosis after circulating and tissue concentrations of the antibiotic fall below effective concentrations). Reestablishment of the dysbiosis leads to chronic inflammation, development of endometritis, and infertility. To test this hypothesis, we designed a study in which cows diagnosed with metritis were matched with healthy control cows at 7 to 10 d postpartum. Subsequently, cows were either treated with systemic antibiotic or were left untreated to create a 2 × 2 factorial arrangement of treatments (metritis-antibiotic, metritis-control, healthy-antibiotic, healthy-control). All cows were slaughtered at approximately one month postpartum when cows were expected to have completed uterine involution and resolved the early postpartum infection of the uterus. The uterus was removed from the abdominal cavity and placed into a biosafety cabinet for sample collection. We found that cows diagnosed with metritis and healthy cows differed in the prevalence of cultured bacteria and 16S rRNA gene sequence reads (greater in cows with metritis). The antibiotic treatment, however, had no effect on the prevalence of cultured bacteria or microbiome. Likewise, the antibiotic treatment had no effect on the prevalence of endometritis in metritic or healthy cows. Regardless of the initial clinical diagnosis (metritis or healthy) or treatment (antibiotic or control), the presence of endometritis at 1 month was associated with specific bacterial species. Given the results of this study and the importance of endometritis to fertility, understanding the mechanisms through which specific bacterial species persist in cows that develop endometritis (regardless of whether they are initially healthy or diagnosed with metritis) is the most important question to be answered in future studies.

## Methods

### Experimental design

**Figure 1:**
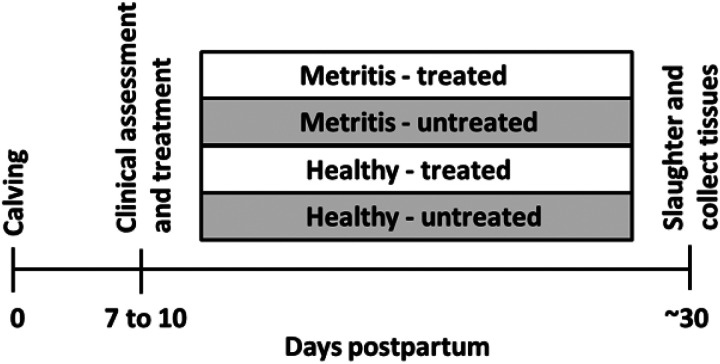
Experimental Design. At d 7 to 10 postpartum, first parity Holstein cows were diagnosed with metritis (fetid red-brown watery vaginal discharge; n=18) or were deemed healthy (normal discharge; n=17) and their uterine contents sampled for 16S rRNA gene sequencing. Immediately after uterine swab collection, cows were either treated with antibiotic [ceftiofur hydrochloride (i.m.; 2.2 mg/kg for 3d); healthy (n = 9), and metritis (n = 9)] or were left untreated [healthy (n = 8), and metritis (n = 9)]. Cows were slaughtered at 29.1±1.7 d postpartum (~30 d postpartum or one month) and tissues processed for bacteriology and metagenomic sequencing.

At 7 to 10 d postpartum, first parity Holstein cows were selected from a confinement herd in eastern Kansas or the MU herd. Cows with a single clinical diagnosis of metritis at 7 to 10 d postpartum (fetid red-brown watery vaginal discharge with a flaccid uterus; (adapted from [24]; n = 18) were selected and matched with clinically healthy postpartum cows [viscous (not watery) and non-fetid discharge; single clinical diagnosis; n = 17] that calved during the same week. The cows enrolled in the study had their uterine contents sampled for 16S rRNA gene sequencing when they were identified and selected for the trial. Uterine swabs were collected transcervically using a double-guarded culture swab (Jorgensen Laboratories, Loveland, CO) following perineal cleaning and disinfection with povidone-iodine. Swab samples were carefully moved to a sterile cryovial (CryoTube Vial; Thermo Fisher Scientific; Waltham, MA) and immediately placed in dry ice, and subsequently stored in a −80°C freezer. Promptly after uterine swab collection, cows were either treated with antibiotic [ceftiofur hydrochloride (i.m.; 2.2 mg/kg for 3 d); healthy (n = 9) and metritis (n = 9)] or were left untreated [healthy (n = 8), and metritis (n = 9); Fig. 1] to create a 2 × 2 factorial arrangement of treatments (metritis-antibiotic, healthy-antibiotic, metritis-untreated control, healthy-untreated control). For cows coming from eastern Kansas, diagnosis was performed within the Kansas herd and cows were moved on the same day to the MU dairy herd, and antibiotic treatment was administered at MU. Cows remained in the MU herd until approximately one month postpartum. The MU herd is a typical dairy confinement herd where cows are housed in a concrete-floored freestall barn and milked twice daily.

Blood samples were collected thrice weekly from the coccygeal veins (10 ml Monoject EDTA, Covidien) and plasma was separated by centrifugation (2,000 ×g for 15 min at 4°C) and stored at −20°C. Plasma haptoglobin concentrations were measured with a commercial ELISA kit by Immunology Consultants Laboratory, Inc. (catalog number E-10HTP, Portland, OR) following the manufacturer’s protocol. Plasma progesterone concentrations prior to slaughter were measured using a validated radioimmunoassay [25].

### Sample collection

Cows were humanely slaughtered by captive bolt stunning and exsanguination at 29.1 ± 1.7 days postpartum. The uterus was removed from the abdomen by transecting the vagina approximately 10 cm from the cervical os. The entire tract was wrapped in a surgical drape, placed on ice, and transported to the microbiology laboratory of the Veterinary Medical Diagnostic Laboratory at the College of Veterinary Medicine at MU. The uterus and ovaries were placed inside a biosafety cabinet and the surgical drape was unwrapped to expose the uterine surface. The presence or absence of a corpus luteum was recorded. The external surface of the uterus (EXT) was sampled for bacteriological culture using a sterile culture swab. Afterward, a tissue sample from the EXT surface near the tip of the horn was collected using sterile forceps and scalpel, inserted into a sterile cryovial, and frozen in liquid nitrogen to be used for 16S rRNA DNA sequencing. The outside of the uterus was then cleaned and disinfected with povidone-iodine and the uterine lumen was exposed by dissection. A sample of the uterine lumen of the previously gravid and non-gravid uterine horns was collected for bacteriological culture and a second sample was collected, placed in a CryoTube, and frozen in liquid nitrogen to be used for 16S rRNA DNA sequencing. Duplicate samples for 16S rRNA DNA sequencing were collected and all samples were immediately snap frozen in liquid nitrogen and then transferred to a −80°C freezer for storage.

Following the collection of samples for bacteriology and 16S rRNA gene sequencing, the reproductive tract was transported to a second laboratory where the uterine lumen was flflushed with 10 mL of cell culture-graded PBS (Gibco, ThermoFisher Scientific). The flflush was recovered into a petri dish and classified visually as either clear (non-purulent, no pus) or containing purulent material (pus). Furthermore, cows with uterine flflush resembling acute metritis (fetid, reddish-brownish discharge) were defined as “acute infection”. Flflush samples were centrifuged at 3000 *x g* at 4°C for 15 min, and the supernatant was collected, homogenized, aliquoted, and stored at −80°C. A longitudinal incision was made along each horn and the lumen of the uterus was inspected visually.

Samples of endometrium were fixed in 10% neutral buffered formalin. The samples were collected from the gravid and the non-gravid horn. Sites for collection were at the mid-point of the horn. The fixed tissues were trimmed to approximately 2 cm of endometrial surface that included a caruncle and inter-caruncular region. A board-certified theriogenologist (T. J. Evans; working independently and blinded to the health status, treatment, or uterine flflush phenotype) scored the amount of inflammation in the uterine epithelium and stroma. Endometrial inflammation [total number of eosinophils (EO), neutrophils, lymphocytes, and plasma cells counted in ten 400X fields from a section of gravid and non-gravid horn] was scored. The inflammation score was 0.5, 1, 1.5, 2, 2.5, and 3 for cows with an average of < 10, 10–19.9, 20–29.9, 30–39.9, 40–49.9, and > 50 cells per field, respectively. Endometrial brosis was also scored as rare (score = 1), rare to occasional (1.5), occasional (score = 2), occasional to frequent (2.5), or frequent (3). Fibrosis for cows scored as 1, 1.5, or 2 always had < 5 layers of fibroblasts. Fibrosis for cows scoring 2.5 or 3 generally had from 5 to 10 layers of fibroblasts.

### Bacterial culture

Tissue samples were ground in a sterile tissue grinder with brain-heart infusion (BHI) broth and inoculated into both solid media and broth for incubation. Swab samples were handled identically except for tissue grinding, and the swabs themselves were incubated in thioglycolate broth. All samples were plated onto tryptic soy agar with 5% sheep blood (TSA), MacConkey agar, phenylethyl alcohol agar (PEA), and thioglycolate broth for incubation under aerobic conditions. Aerobic cultures were incubated at 36°C in a standard ambient air incubator. Capnophilic cultures were maintained at 36°C under 5% CO2. Campylobacter cultures were placed in Mitsubishi boxes equipped with a microaerophilic sachet (Mitsubishi AnaeroPak MicroAero gas generator, Remel), providing 6–12% O2 and 5–8% CO2, and then incubated at 42°C for enteric *Campylobacter* and 35°C for reproductive *Campylobacter*. Samples were also plated onto TSA and PEA for incubation under anaerobic conditions; chocolate agar, Hayflick agar, and BHI broth for incubation under 5% CO2; and selective *Campylobacter* agar for incubation under microaerophilic conditions. Anaerobic cultures were held in Mitsubishi boxes using a Mitsubishi AnaeroPack anaerobic gas generator (Remel; Lenexa, KS), and anaerobic conditions (< 1% oxygen, > 15% CO2) were verified using anaerobic indicators (Remel; Lenexa, KS). All bacterial culture media used were sourced from Remel (Lenexa, KS), except for the reproductive *Campylobacter* medium, obtained pre-reduced from Anaerobe Systems (Morgan Hill, CA). Media were incubated for 7 days and evaluated daily. All isolates were identified via MALDI-TOF mass spectrometry, standard biochemical tests, and/or 16S rRNA sequencing.

### 16S rRNA gene sequencing (metagenomics)

A manual precipitation protocol was used for DNA extraction [26]. Library construction and sequencing were performed by the University of Missouri DNA Core. A Qubit dsDNA BR Assay (Life Technologies, Carlsbad, CA) was used to determine DNA concentration. Samples were normalized to 3.51 ng/μL DNA for PCR amplification. The V4 hypervariable region of the 16S rRNA gene was amplified using single-indexed universal primers [U515F (GTGCCAGCMGCCGCGGTAA); 806R (GGACTACHVGGGTWTCTAAT)] with standard adapter sequences (Illumina Inc., San Diego, CA). The PCR program for amplification was: 98°C (3:00) + [98°C (0:15) + 50°C (0:30) + 72°C (0:30)] × 40 cycles + 72°C (7:00; min:s). The V4 region of the 16S rRNA gene was selected for library generation because this region yields optimal community clustering [27]. The Illumina MiSeq platform (V2 chemistry with 2 × 250-bp paired-end reads) was used to sequence pooled amplicons.

### Analysis of 16S rRNA gene sequences

Amplicon sequences from the V4 hypervariable region of the 16S rRNA gene were processed and analyzed using QIIME2 (version 2020.6, https://qiime2.org) [28]. Fastq files containing forward and reverse sequences were imported into QIIME2 and demultiplexed to assign sequences to samples. The plugin cutadapt [29] was used to trim off PCR primers (515F/806R) from raw sequences. QIIME2 Divisive Amplicon Denoising Algorithm (DADA2) plugin was used for detecting and correcting Illumina amplicon sequencing errors [30]. QIIME2 quality-control plugin was used to exclude contaminant sequences such as host sequences (e.g., cow DNA) and non-targeted (e.g., non-bacterial) sequences. Green Genes (https://greengenes.secondgenome.com) operational taxonomic unit (OTU) reference sequences (99% sequence identity) were used for quality control. Sequences filtered out during this step were investigated using the NCBI BLAST nucleotide database (https://blast.ncbi.nlm.nih.gov/Blast.cgi) to ensure that only contaminant sequences were removed. One hundred and twenty-three contaminant sequences were eliminated in this process (Supplemental Dataset S1). Sequences filtered out during this step were investigated using the National Center for Biotechnology Information (NCBI) blast nucleotide database (Supplemental Dataset S2). Predicted matching eliminated sequences included regions in bovine, fungal, and viral genomes among others.

To perform phylogenetic diversity analyses, a rooted phylogenetic tree was generated using the QIIME2 phylogeny function after samples were rarefied to depths of 15,000 and 1,000 sequences for the experiments performed during the first week postpartum (e.g., uterine swab sample) or first month (low biomass environment) postpartum (samples collected at animal slaughter), respectively. Pairwise comparisons for alpha diversity measures (Pielou’s Evenness [31] and Faith’s Phylogenetic Diversity [32]) were computed using the Kruskal-Wallis test. The unweighted UniFrac distances, a measure of beta diversity [33, 34] were also calculated and PERMANOVA was used in pairwise comparisons to evaluate beta-diversity group distances. Furthermore, principal coordinate analyses (PCoA) plots for qualitative (presence/absence; Jaccard distances [35]) and quantitative (abundance and presence/absence; Bray-Curtis [36]) metrics were generated using Emperor [37, 38] to aid in data visualization and interpretation.

A pre-formatted taxonomy classifier [39] was used for assigning taxonomy classification to the 16S rRNA amplicon sequences [40–43], and an amplicon sequence variant (ASV) table was generated [44]. Amplicon sequence variants sharing the same taxa were collapsed together (at the species level) using the QIIME2 taxa collapse function.

Differential abundance analysis on the identified ASV was performed using the Analysis of Composition of Microbes (ANCOM) statistical framework [45]. For ANCOM, data was pre-processed to remove features with low reads (less than 10 reads across all samples), rarely observed (present in less than 2 samples), and with low variance (less than 10e-4). Because ANCOM is based on log ratios, QIIME2 add-pseudocount plugin was used to add one count to every feature, allowing ANCOM analysis to be performed on features with zero counts. Pairwise comparisons using the ANCOM framework were conducted to compare the microbiome of samples from different health statuses (*e.g*., metritis versus healthy at clinical diagnosis; and clear versus purulent flflush at one month postpartum). The Qiime2 EMPress plugin [46] was used to generate a phylogenetic tree for exploring the hierarchical evolutionary relationships of features subjected to ANCOM.

Principal coordinate and diversity analyses indicated that the uterine microbiome at 30 d postpartum was strongly associated with the visual characteristics of the uterine fluid (clear, purulent, or acute metritis) which was evaluated at the time of sample collection. A multinomial regression analysis was conducted using Songbird [47] to investigate differences in microbial composition within the uterine lumen which were associated with the uterine flflush phenotypes (clear, purulent, or acute metritis) while accounting for important factors, such as whether or not cows had postpartum metritis (healthy versus metritis) and the antibiotic treatment regimen (treated versus not treated) that each cow was subjected to at enrollment (day 7 to 10 postpartum).

Because the initial microbial load of individual samples submitted for sequencing was unknown in the present study, the analytical approach implemented by Songbird [47] was based on ranking microbes relative to each other within a sample. The main output of this analysis is a file containing the “differentials”, which are the logarithm of the fold change in abundance of taxa between two conditions. Relative differentials were estimated using a multinomial regression, and the coefficients from the multinomial regression were ranked to determine which taxa changed the most within samples and across conditions. Three distinct models were generated using the standalone version of Songbird. The first model, termed “null model”, was created to represent random chance (this model was run without any metadata). The null model was used for comparison to evaluate whether additional models containing selected variables presumed to be associated with uterine disease at 30 d postpartum were more predictive than random chance. The second model contained only the categorical information of flflush phenotype, as a clear uterine fluid is an evident sign of a healthy uterus, a flflush containing purulent material is indicative of infection, and a flflush with fluid resembling early postpartum metritis (acute disease) is strong evidence of severe infection. Lastly, a third model, termed “full model”, was created containing variables representing the flflush phenotype (clear, purulent, or acute), cow identification, disease status one week postpartum (healthy versus metritis), antibiotic therapy (treated versus not treated), and plasma progesterone concentrations at the time of slaughter (indicative of cyclicity). Outputs from the three models were evaluated using TensorBoard, a visualization toolkit of Tensorflow [48], an open-source machine learning library developed by the Google Brain team (Mountain View, CA). TensorBoard provides measurements and visualizations needed for a machine learning workflow, including metrics such as loss and accuracy that were carefully evaluated for each model tested. Furthermore, the interactive web application Qurro [49] was used to plot the differentials generated in Songbird.

Songbird was also run as a QIIME2 plugin, and the pseudo-Q2 score was calculated for the two models tested against the null model. The pseudo-Q2 score indicates the predictive accuracy of the model. It is calculated using the formula Q^2^ = 1 – m1/m2, where m1 indicates the average absolute error for the testing model and m2 indicates the average absolute error for the null model (representing random chance). A pseudo-Q2 score close to 1 indicates high predictive accuracy of the testing model, whereas a pseudo-Q2 score that is low or below zero indicates poor predictive accuracy and possible overfitting.

### Statistical analyses performed using SAS

Descriptive continuous data representing physiological parameters measured from the study cows (*e.g*., rectal temperature, circulating haptoglobin concentrations, inflammation score, etc.) and the sum of 16S rRNA gene reads identified per cow according to disease classification (*e.g*., Healthy versus Metritis), flflush phenotype (*e.g*., clear, purulent, and acute) or disease by antibiotic treatment classification (e.g., Metritis-antibiotic, Metritis- untreated, Healthy-antibiotic, Healthy-untreated,) were analyzed using a factorial design with mixed models procedures of SAS 9.4 (PROC MIXED; SAS Institute Inc., Cary, NC). Prior to analyses, all continuous data mentioned was assessed for normality using the Univariate procedure of SAS. Statistical significance was defined as *P* ≤ 0.05 and statistical tendencies as 0.05 < *P* < 0.10.

## Results

### Clinical diagnosis of metritis

**Figure 2:**
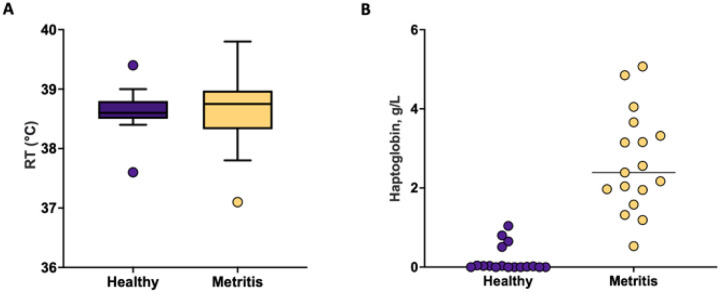
Rectal temperature (*P*= 0.91) **(A)** and plasma concentrations of haptoglobin (*P* < 0.01) **(B)** measured at the time of disease diagnosis (healthy or metritis; 7 to 10 days postpartum).

Rectal temperatures (P = 0.91) were similar (Fig. 2A) but plasma concentrations of acute-phase protein haptoglobin (Fig. 2B) were greater (*P* < 0.01) for metritis (38.7 ± 0.1 °C and 2.65 ± 0.23 g/L) compared with healthy (38.6 ± 0.1 °C and 0.19 ± 0.23 g/L) cows at time of disease diagnosis (7 to 10 d postpartum).

### Uterine microbiome at disease diagnosis

**Figure 3:**
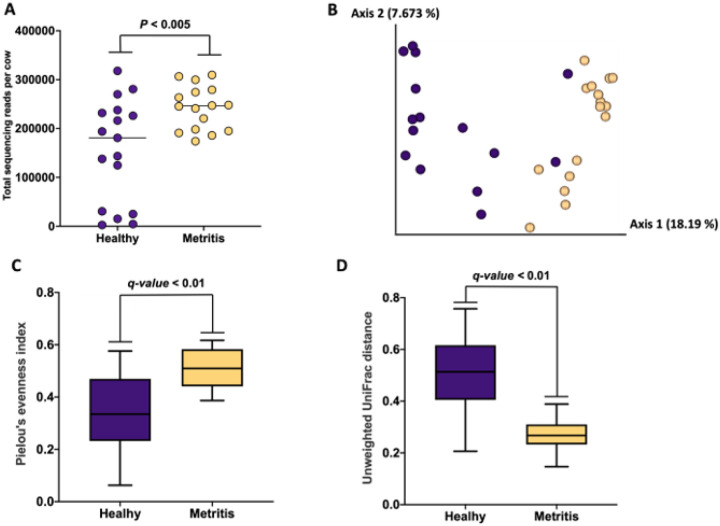
Sum of sequencing reads per cow according to disease status (Healthy vs Metritis) **(A)**, Jaccard principal coordinate analysis (PCoA) plot **(B)**, Pielou’s index **(C)**, and unweighted Unifrac distances **(D)** from uterine swabs collected from metritis (blue symbols and bars) and healthy (yellow symbols and bars) cows at the time of disease diagnosis. Of note, swab samples were not collected for two cows diagnosed with metritis.

There were a total of 541 ASV detected in uterine swab samples collected at the time of metritis diagnosis. Cows that had been diagnosed with metritis had a greater overall number of sequencing reads (*P* < 0.005) when compared with healthy cows (Fig. 3A). The Jaccard and Bray-Curtis PCoA plots demonstrated differences in microbial composition between metritis and healthy cows as evidenced by the clustering of individual cows from the respective groups (Fig. 3B and Supplemental Fig. 1, respectively). The Pielou’s index was greater in metritis compared with healthy cows (q-value < 0.01; Fig. 3C) indicating that the abundance of ASV detected in cows with metritis was more even (present in similar proportions) compared with ASV detected in healthy cows. In contrast, the unweighted Unifrac distance was greater (q-value < 0.01) in healthy compared with metritis cows (Fig. 3D). This result indicates that the microbiome of metritis cows was more well-defined (less diverse) than the microbiome of healthy cows.

**Figure 4:**
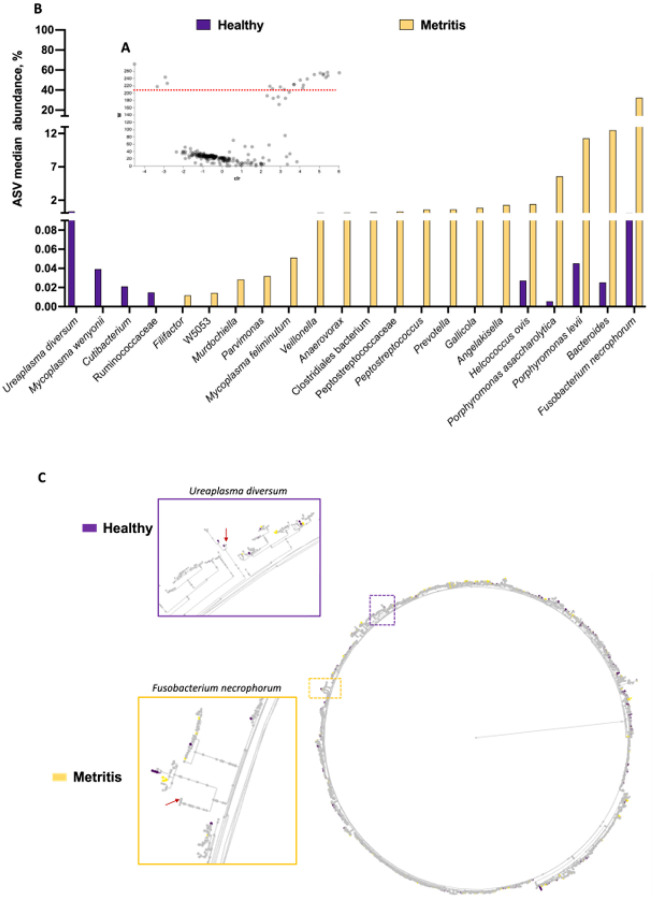
Differential abundance analysis with ANCOM for microbiomes collected from uterine swabs performed at the time of disease diagnosis. Volcano plot **(A)** highlighting the 22 differently abundant ASV (18 increased and 4 decreased in metritis compared with healthy cows). The red dotted line represents the cut-off for the W-value (y-axis) indicating statistical significance (features above the dotted line were significantly different between metritis and healthy cows). The W-value represents a count for the number of times that the null hypothesis was rejected for a given ASV. The null hypothesis states that the average abundance of a given ASV is equal in metritis and healthy cows. The x-axis displays the centered log ratio (clr) of the abundance of a given ASV between healthy and metritis cows. A negative clr means an ASV (each gray dot in the volcano plot) is more abundant in healthy than metritis cows, and a positive clr means that the ASV is more abundant in the metritis compared to healthy cows. There were 6 ASV that approached significance tending to increase in metritis compared with healthy cows (*Filifactor, Peptococcus simiae*, SR1 bacterium, *Clostridium cadaveris, Tissierella* and *Helcococcus*). **(B)** Histogram plotting the median abundance of ASV that were significantly different between healthy and metritis cows based on ANCOM. The displayed abundances (y-axis) were calculated as the median for the percentage of reads mapped to each ASV of interest, out of the total reads sequenced per cow. **(C)** A phylogenetic tree from EMPress [46] showing the hierarchical evolutionary relationships for the features subjected to ANCOM. The outermost tips of the tree are termed “leaves” and represent a unique ASV. The purple and yellow dotted squares represent areas of the phylogenetic tree that were zoomed in. The red arrows point to the leaves representing the *Ureaplasma diversum* (purple square) and *Fusobacterium necrophorum* (yellow square), the most differently abundant ASV that increased in healthy and metritis cows, respectively. Importantly, gray leaves represent ASV that were identified in both healthy and metritis cows, whereas leaves that are purple or yellow represent ASV that were uniquely observed in either healthy or metritis cows, respectively.

Results from the differential abundance analysis using ANCOM are summarized in Figs. 4A and 4B. There were 22 differentially abundant ASV identified by ANCOM (Fig. 4A). From the 22 differently abundant features, 18 were increased in the uterus of metritis cows whereas 4 were increased in healthy cows. There were six additional ASV that approached significance (W-values ranging from 168–202) tended to be increased in metritis compared with healthy cows (*Filifactor, Peptococcus simiae, SR1 bacterium, Clostridium cadaveris, Tissierella, and Helcococcus*; Supplemental Table S1). The abundance of significantly different ASV between healthy and metritis cows according to ANCOM are depicted in a histogram (Fig. 4B). The dysbiosis associated with metritis was marked by a significant change in the microbial community for only a small population of bacteria (~ 28 out of 541 detected ASV).

A phylogenetic tree was generated using EMPress [46] to depict the hierarchical evolutionary relationships for the features subjected to ANCOM (Fig. 4C). Gray leaves represent ASV that were identified in both healthy and metritis cows, whereas leaves that are colored purple or yellow represent ASV that were uniquely observed in either healthy or metritis cows, respectively. The majority of the ASV identified were present in both healthy and metritic cows (most of the leaves in the phylogenetic tree were gray) with few purple and yellow leaves spread out across the tree. These findings support the idea that dysbiosis in metritis is mainly associated with the overgrowth of a small population of bacteria relative to control.

#### Gross morphological observations and histological analyses of tissue at slaughter (one month postpartum).

Based on analyses of thrice-weekly plasma progesterone, there were 3 out of 18 metritis cows and 7 out of 17 healthy cows cycling (> 1 ng/mL plasma progesterone) prior to slaughter. There were 20 (57%), 12 (34%) and 3 (9%) cows that were defined as having a clear, purulent, or acutely infected uterine lumen (Table 1). Among cows diagnosed as healthy at 7 to 10 d postpartum (n = 17), there were 5 cows (29%) that had a purulent flflush at slaughter. For metritis cows (n = 18), there were 10 cows (56%) that were either purulent or acutely infected at slaughter. The antibiotic treatment did not change the proportion of purulent or purulent/acutely infected cows within the healthy or metritis groups (Table 1). There was no effect of the original disease diagnosis on the uterine histological score for fibrosis, epithelial inflammation, or stromal inflammation (Table 2). The eosinophil score was greater (*P* < 0.008) for metritis versus healthy cows. Fibrosis score (*P* < 0.006), epithelial inflammation score (*P* < 0.001), stromal inflammation score (*P* < 0.004), and eosinophil score (*P* < 0.001) were greater for cows with a purulent flflush compared with a clear flflush. We did detect a limited number of interactions of antibiotic treatment with main effects but these were generally small in magnitude (Table 2).

#### Bacterial culture of tissue at slaughter (one month postpartum).

A complete list of cultured bacteria from all locations (external surface, gravid horn, and non-gravid horn) is provided (Supplementary Table 2). There were a large number of bacteria isolated from the external surface of the reproductive tract (contamination that arose during tissue collection; Table 3) and every individual cow had bacterial growth. There was no effect (*P* > 0.10) of the original disease diagnosis or the antibiotic treatment on the average prevalence (percentage of cows with a particular species) of the major species found on the external surface of the tract. Within the lumen of the uterine horn, there was no growth for 8 of 17 (47%) healthy cows and 3 of 18 (17%) metritis cows. For the most-prevalent species (Table 4), cows with early postpartum metritis had a greater prevalence (12.0 ± 1.3%) compared with healthy cows (1.2 ± 1.3%) (*P* < 0.001) and there was no effect (*P* = 0.189) of antibiotic treatment on the average prevalence of the listed species (Table 4).

**Figure 5:**
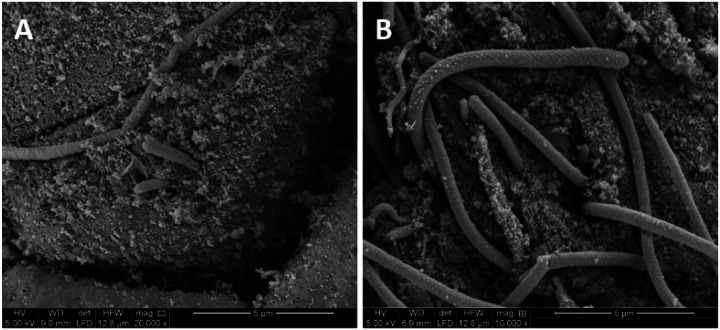
Scanning electron micrograph of the uterine surface of a cow classified as acutely infected at 30 d postpartum. The long cylindrical organisms inserted into the cells on the surface of the uterus have a morphology that is consistent with *Fusobacterium necrophorum* [50]. Bacterial culture also identified *Fusobacterium necrophorum* in the uterine lumen of this cow.

Forty-five percent of cows with a clear uterine flflush had no growth on bacterial culture compared with 17% and 0% for cows with a purulent lumen or an acute infection (Table 5). All three cows with an acute infection were infected with *Fusobacterium necrophorum* and *Trueperella pyogenes*. Scanning electron microscopy of the surface of the uterus of a cow with acute uterine infection demonstrated the presence of bacteria with morphology consistent with *Fusobacterium necrophorum* (Fig. 5 [50]). Other cultured species with a high prevalence among cows with an acute infection or a purulent lumen were *Helcococcus ovis, Histophilus somni, Peptoniphilus indolicus, Porphyromonas levii*, and *Streptococcus pluranimalium*.

#### Effect of location on the uterine microbiome at tissue collection.

**Figure 6:**
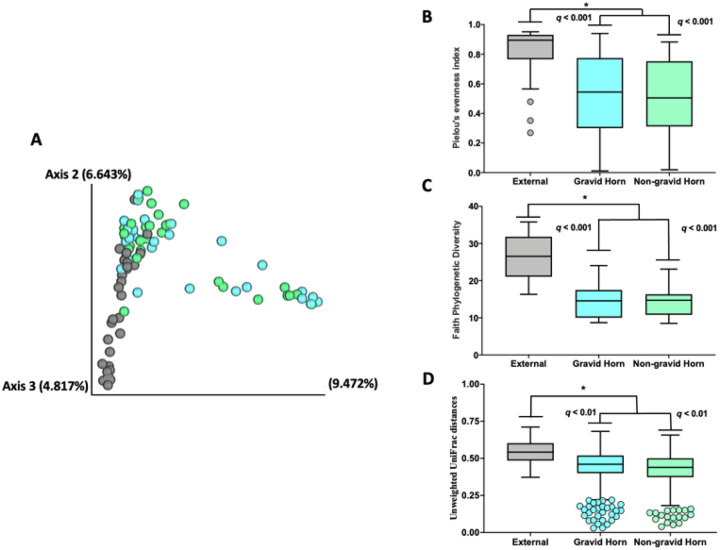
Jaccard PCoA plot **(A)**, Pielou’s evenness **(B)**, Faith Phylogenetic Diversity **(C)** and Unweighted Unifrac distances **(D)** for the microbial composition between samples collect at approximately one month postpartum from the previously gravid (light blue bars and symbols) and non-gravid (light green bars and symbols) uterine horns compared with external control samples collected from the outside of the uterus (gray bars symbols). Individual datapoints plotted beyond the box-and-whiskers plots represent outliers according to the 1.5 interquartile range (IQR) method.

There were 1,560 ASV detected in the uterine horns and control samples following tissue collection one month postpartum. The Jaccard and Bray-Curtis PCoA plots (Fig. 6A and Supplemental Figure S2, respectively) demonstrated similarities in microbial composition between samples from the previously gravid (light blue symbols) and non-gravid (light green symbols) horns, and differences in the microbial composition of samples from the uterine horns compared with external control samples collected from the outside of the uterus (gray symbols). The Pielou’s evenness (Fig. 6B) was increased in control external samples compared with samples collected from the lumens of the gravid and non-gravid horns (q-value < 0.01), but was not different between the gravid and non-gravid horns (q-value = 0.63). Similarly, Faith’s Phylogenetic Diversity (Fig. 6C), another alpha diversity measure that incorporates phylogenetic differences between species [32] was also increased in the external control samples compared with samples collected from the lumens of the gravid and non-gravid horns (q-value < 0.01) but not different between the gravid and non-gravid horns (q-value = 0.63). The unweighted Unifrac distances (Fig. 6D) among external control samples were also increased compared with samples from the lumens of the gravid and non-gravid horns (q-value < 0.01) but not different between the gravid and non-gravid horns (q-value = 0.98).

**Figure 7:**
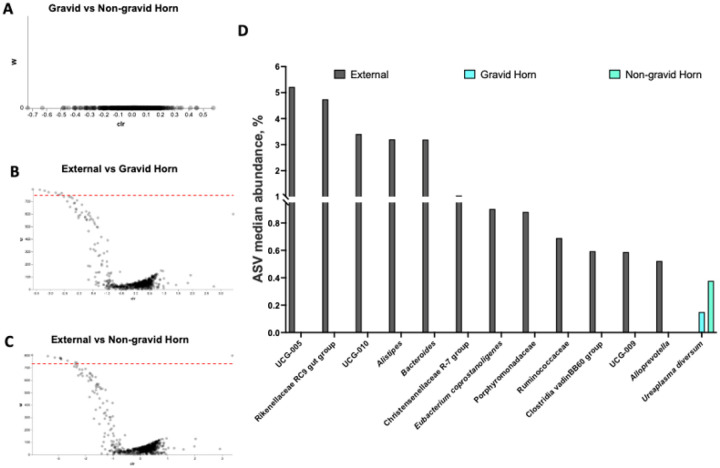
Results from the differential abundant analyses with ANCOM among samples from control, gravid, and non-gravid uterine horns. There were no differences in microbial composition between the previously gravid and non-gravid uterine horns **(A)**. The microbial composition within the lumen of the previously gravid **(B)** and non-gravid **(C)** uterine horns differed from that of external control samples for 12 and 12 ASV for the gravid and non-gravid horns respectively (ASV above the dotted red significance line in the volcano plot). Overall, among ASV that were differently abundant within the lumen of the uterus, the abundance of 12 bacteria increased on the outside of the tract and one bacterium (*Ureaplasma diversum*) increased in the lumen of the uterine horns **(D)**.

Results from the differential abundance analyses with ANCOM among samples from control, gravid, and non-gravid uterine horns are summarized in Figs. 7A to 7D. There were no differences in microbial composition between the previously gravid and non-gravid uterine horns (Fig. 7A). Conversely, the microbial composition within the lumens of the previously gravid and non-gravid uterine horns differed from that of external control samples (Figs. 7B and 7C, respectively). The abundance of 12 ASV significantly increased in external control compared with the previously gravid horn samples (Fig. 7B, Supplemental Table S3), and the abundance of 11 ASV increased and 1 ASV decreased in samples from the lumen of the non-gravid horn compared with external control samples (Fig. 7C; Supplemental Table S4). Overall, among ASV that were differently abundant within the lumen of the uterus (previously gravid and non-gravid horns combined) and the outside of the tract (external control sample) after collapsing ASV representing the same bacteria, the abundance of 12 bacteria was increased on the outside of the tract and one bacterium (*Ureaplasma diversum*) was increased in the lumens of the uterine horns (Fig. 7D).

#### Effect of antibiotic treatment and original disease diagnosis on uterine microbiome at tissue collection (one month postpartum).

**Figure 8:**
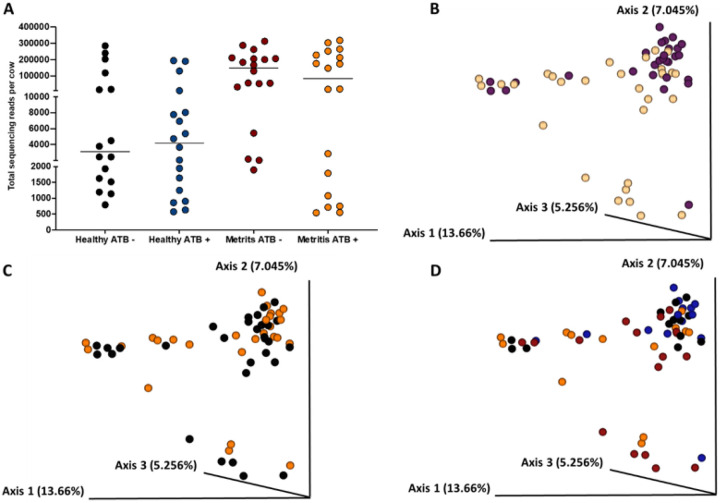
Uterine microbiome of cows that had been diagnosed at 7 to 10 d postpartum (as metritic or healthy controls) and had their uterine microbiome sampled and analyzed after tissue collection at one month postpartum. Number of 16S rRNA sequencing reads per cow **(A)**, the Jaccard PCoA plot **(B)** for cows that were either healthy (purple dots) or metritis (yellow dots) at disease diagnosis, the Jaccard PCoA plot **(C)** for cows that were either antibiotic-treated (orange dots) or untreated (black dots) at disease diagnosis, and the Jaccard PCoA plot **(D)** for the interaction of disease diagnosis and treatment (Healthy-antibiotic, blue dots; Healthy-not treated, black dots; Metritis-antibiotic treated, orange dots; Metritis-not treated, red dots) are shown.

Data from both the gravid and the non-gravid horns were analyzed collectively to evaluate the effects of systemic antibiotic treatment during the first week postpartum on the uterine microbial composition of metritis and healthy cows at one month postpartum. Cows that had been diagnosed as healthy one week postpartum had fewer sequencing reads in samples collected at one month postpartum (*P* < 0.003) compared with metritis cows (Fig. 8A). The Jaccard and Bray-Curtis PCoA plots (Fig. 8B and Supplemental Figure S3A) demonstrated that the uterine microbiome of cows that were healthy at disease diagnosis generally clustered together in the PCoA plots at one-month postpartum. There was more variability (individual data points more spread apart within the PCoA) in the microbiome of cows that had been diagnosed with metritis around the first week postpartum, indicating a less uniform microbial population across all metritis cows at one month postpartum (Fig. 8B and Supplemental Figure S3A). Additionally, alpha diversity metrics in uterine samples collected one month postpartum, including Pielou’s evenness (q-value = 0.01) and Faith’s PD (q-value = 0.06), were greater or tended to be greater in healthy cows compared with metritis cows, respectively. Conversely, the unweighted Unifrac distances of samples collected one month postpartum were increased (q-value = 0.004) in metritis compared with healthy cows. According to ANCOM, the only differently abundant feature between healthy and metritis cows at day 30 was from the genus *Cutibacterium*, which had increased abundance in the uterine lumen of healthy compared to metritis cows.

The antibiotic treatment at disease diagnosis did not affect (*P* = 0.48) the number of 16S rRNA gene reads in uterine samples collected one-month postpartum (Fig. 8A), nor the alpha (Pielou’s evenness q-value = 0.25; Faith’s PD, q-value = 0.86) or beta (unweighted Unifrac distance q-value = 0.54) diversity metrics evaluated (data not shown). Furthermore, antibiotic treatment did not affect the microbiome composition of the uterus at one month postpartum, as samples from treated cows (orange) or untreated cows (black) did not separate in the Jaccard or Bray-Curtis PCoA plots (Fig. 8C and Supplemental Figure S3B, respectively). Based on ANCOM, there were no differently abundant features in uterine samples collected at one month postpartum for antibiotic-treated and untreated cows. The interactions of uterine disease (healthy versus metritis) with antibiotic treatment (treated versus untreated) on the microbiome composition of the uterus one month after calving are shown with the Jaccard and Bray-Curtis PCoA plots, Figs. 8D and Supplemental Figure S3C, respectively.

#### Uterine microbiome of cows with a clear, purulent, or acutely infected uterine lumen at tissue collection (one month postpartum).

**Figure 9:**
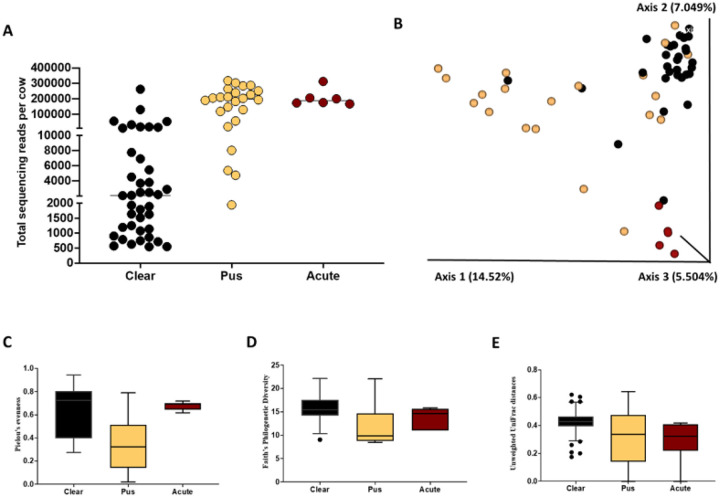
Total number of sequencing reads per sample **(A)**, Jaccard PCoA plot **(B)**, Pielou’s evenness (**C**), Faith’s Phylogenic diversity (**D**), and Unweighted UniFrac distances (**E**) from the uterine horns (previous gravid and non-gravid combined) at tissue collection (one month postpartum) for cows with a clear or purulent or acutely infected uterine flush.

The sum of sequencing reads obtained per sample from the uterine horns (previous gravid and non-gravid combined) at tissue collection (one month postpartum) was greater (*P* < 0.01) in cows with evidence of infection (purulent or acutely infected) compared with cows with a clear flflush (Fig. 9A). The Jaccard and Bray-Curtis PCoA plots (Fig. 9B and Supplemental Figure S4, respectively) demonstrated distinct uterine microbial composition in cows with diverging uterine flflush phenotypes.

With respect to the alpha diversity measures, Pielou’s evenness was decreased in cows with purulent compared with clear flflush (q-value < 0.01) or acute infection (q-value < 0.01) (Fig. 9C). Pielou’s evenness was not different between cows with clear flflush and acute infection (q-value = 0.84; Fig. 9C). Faith’s PD was also decreased (q-value < 0.01) in cows with purulent compared with clear uterine flflush, but no differences (q-value = 0.13) were detected for the remaining pairwise comparisons (Fig. 9D). For beta diversity, the unweighted (qualitative; presence/absence) UniFrac distances among samples from cows with clear flflush were greater (q-value = 0.001) than in samples from cows with purulent flflush and acute infection (Fig. 9E). Furthermore, the unweighted UniFrac distances were also greater (q-value = 0.001) among samples of cows with purulent compared with cows with acute infection (Fig. 9E).

#### Analysis of the uterine microbiome one month postpartum using a multinomial regression.

**Figure 10:**
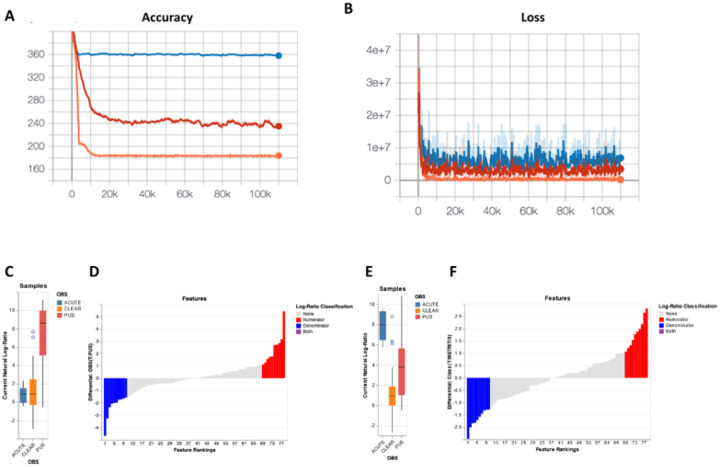
Multinomial regression analysis of the uterine microbiome 30 days postpartum. (**A**) Graph representing the prediction accuracy (capacity of the model to predict the observed read counts in samples) for the null model (blue line), a model with only the categorical information of flush phenotype (red line) and the full model (orange line). The x-axis shows the number of iterations which represents the number of times the model trained across the entire dataset. The y-axis shows the cross-validation (CV) values representing the accuracy of the model. (**B**) The loss graph represents how well each model fits the data. The y-axis is the negative log probability of the model fitting the data (a lower number indicates a better fit of the model). The x-axis indicates the number of iterations. The “sample plot”, (**C and E**), shows the log-ratios of abundance within samples for the selected top 10 ASV that increased and decreased (selected top 10 and bottom 10 ASV) in cows with purulent and acute flush phenotype compared with cows with clear phenotype, respectively. Furthermore, the “rank plot”, (**D and F**), shows the feature rankings, a visualization of how features are ranked in comparison with the other features evaluated.

Outputs from the three models generated in Songbird were evaluated using TensorBoard (Figs. 10A and 10B). The prediction accuracy (capacity of the model to predict the observed read counts in samples) for each model is depicted in Fig. 10A. The null model, the blue line, represents random chance. The model with only the categorical information of flflush phenotype (clear, purulent, or acute infection) is represented by the red line. The complete “full model” includes the information of flflush phenotype, cow identification, disease status at 7 to 10 d postpartum (healthy vs metritis), antibiotic treatment (yes or no), and plasma progesterone concentrations at the time of slaughter (indicative of cyclicity) represented by the orange line. The x-axis represents the number of iterations which indicates the number of times the model is training across the entire dataset. The y-axis constitutes the cross-validation (CV) values representing the accuracy of the model. For instance, the full model (orange line) is off by, on average, 170 counts, while the null model is off by 360 counts.

The loss function (Fig. 10B) represents how well each model fits the data. The y-axis is the negative log probability of the model fitting the data (a lower number indicates a better fit of the model). The x-axis indicates the number of iterations. The full model (orange line) is closer to 0, indicating a better fit. Overall, considering the accuracy and loss graphs, the full model significantly outperformed the null model and the model containing only the information of flflush phenotype. The pseudo-Q2 scores generated by Songbird through QIIME2 further validate this observation: null model versus full model (Q^2^ = 0.49); null model versus flflush phenotype only (Q^2^ = 0.33). A pseudo-Q2 score close to 1 indicates high predictive accuracy of the testing model, whereas a pseudo-Q2 score that is low or below zero indicates poor predictive accuracy, and possibly overfitting.

**Figure 11:**
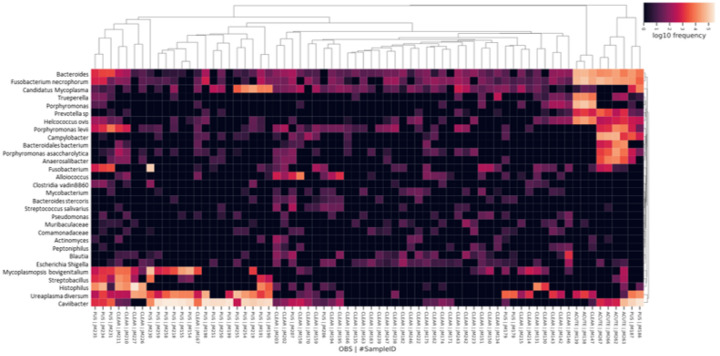
Heatmap highlighting the most differently abundant ASV among cows with clear, purulent (pus), or acutely infected flush phenotype at 30 days postpartum based on multinomial regression performed using Songbird. The top 10 ASV that increased or decreased in cows with purulent (pus) compared with cows with clear uterine flush phenotype, and the top 10 ASV that increased or decreased in cows with acute compared with cows with clear flush phenotype are presented in the heatmap. The Linnaean classification system was used for labeling the taxa presented in the heatmap. If available, the species’ name was shown. Otherwise, broader terms were used as needed for ASV identification.

Relative differentials were estimated from the full model to determine which taxa changed the most between samples and across conditions [47]. The estimated log-fold change in abundance of the detected features was visualized using Qurro [49]. The “sample plot”, Figs. 10C and 10E, shows the log-ratios of abundance within samples for the selected top 10 ASV that increased and decreased (selected top 10 and bottom 10 ASV) in cows with purulent and acute flflush phenotype compared with cows with clear phenotype, respectively. Furthermore, the “rank plot”, Figs. 10D and 10F, shows the feature rankings, a visualization of how features are ranked in comparison with the other features evaluated. Of note, there were a total of 78 features (ASV) ranked out of 1,560 features detected at one month postpartum (Fig. 10C). This was because ASV present in less than 10 samples, and samples with less than 1,000 reads were filtered out to improve the linear regression during the Songbird workflow. The top 10 ASV that increased or decreased in cows with purulent compared with cows with clear flflush phenotype are presented in Supplementary Table S5. Similarly, the top 10 ASV that increased or decreased in cows with acute compared to cows with clear flflush phenotype are presented in Supplementary Table S6. A heatmap for the top 30 most differently abundant features according to Songbird (Supplementary Tables S5 and S6) is presented in Fig. 11.

## Discussion

This study tested whether systemic treatment with antibiotics early postpartum changed the progression of uterine disease through a sustained change in the uterine microbiome. Primiparous Holstein cows were used to avoid confounding effects of previous pregnancies. Cows diagnosed with metritis at 7 to 10 d postpartum were matched with healthy controls and each group was randomly assigned to receive antibiotic or no treatment (2 by 2 factorial design). Cows were slaughtered at about one month after calving to assess the inflammatory status of the uterus and uterine microbiome. We performed traditional bacteriological culture to assess whether there were viable organisms inside the reproductive tract and also 16S rRNA gene sequencing to assess the entire microbiome.

Metritis and healthy cows were distinctly different at disease diagnosis (7 to 10 d postpartum). Cows diagnosed with metritis had elevated plasma haptoglobin concentrations (Fig. 2B) compared with controls. Haptoglobin is an acute-phase protein that is typically increased in cows with metritis. According to the data of Huzzey et al. [51] haptoglobin above 1 g/L and below 0.5 g/L within 1 week postpartum would indicate metritis and healthy diagnosis, respectively. We clearly achieved the thresholds in this study (Fig. 2B). We also found that the uterine microbiome at disease diagnosis was distinctly different for metritis versus healthy cows (Figs. 3 and 4). Individual cows evidently differed on the Jaccard PCoA plot (Fig. 3B) for the respective groups. The Pielou’s index, an alpha diversity metric for community evenness, was greater in metritis compared with healthy cows (q-value < 0.01; Fig. 3C) indicating that the abundance of ASV detected in metritis cows was more even (present in similar proportions) compared with ASV detected in healthy cows. In contrast, the unweighted Unifrac distance, a measure of beta diversity, was greater (q-value < 0.01) in healthy compared with metritis cows (Fig. 3D). The unweighted Unifrac distance is a qualitative measure, meaning that it uses the presence/absence of ASV as opposed to the abundance to compare community composition, in addition to accounting for phylogenetic relationships among the detected features [34]. This result indicates that the microbiome of metritis cows was more well-defined (less diverse) than the microbiome of healthy cows. The greater evenness and lesser diversity that we found in cows diagnosed with metritis typify the “dysbiosis” that is often described in acute infectious diseases like metritis.

The microbiome of cows with metritis was dominated by bacterial species previously associated with metritis including *Fusobacterium necrophorum, Bacteroides, Porphyromonas levii* and *Helcococcus ovis* [14] (Fig. 4B). We also noted bacteria with greater abundance in healthy cows at disease diagnosis including *Ureaplasma diversum, Mycoplasma wenyonii*, and *Cutibacterium*. When we examined the broader microbial community (Fig. 4C) we noted that the bulk of sequenced organisms were similarly represented in metritis versus healthy cows. Indeed, at disease diagnosis (d 7 to 10 postpartum) we detected only approximately 5% (28 out of 541) ASV that were differentially abundant between metritis and healthy control.

Cows were treated and then allowed to reside in the herd as lactating cows until approximately 1 month postpartum. At that time, cows were slaughtered and tissues were collected for visual inspection, bacterial culture, and 16S rRNA gene sequencing. Fifteen cows had purulent material in the uterus or had an acute infection (Table 1). Purulent material in the uterus or acute infection was associated with more uterine inflammation (evidence of endometritis) compared with a clear uterine flflush (Table 2). We noted that 5 of the healthy cows (29%) had purulent material (pus) in the uterus at slaughter. We also noted that there were 8 (44%) cows that had been originally diagnosed with metritis (7–10 d postpartum) whose uterine lumen was entirely clear one month postpartum (Table 1). This high rate of “self-cure” from metritis is consistent with published work [20]. Although we could diagnose metritis early postpartum on the farm, this initial diagnosis was not necessarily associated with a clinical diagnosis of endometritis (inflammation and pus in the uterus) at one month postpartum. The number of cows that crossed over (healthy to diseased or vice-versa) was large (29 and 44% in our study) and this likely complicates our ability to fully understand the effect of metritis on cow health and subsequent fertility. We also do not fully understand whether the initial diagnosis of metritis or the subsequent diagnosis of endometritis or both is needed to account for the effects of disease on fertility of postpartum cows. Of interest was the additional observation that the number of cows that crossed over did not appear to be affected by antibiotic treatment (Table 1) with the caveat that the study was not adequately powered to test this question.

We examined the microbiome of cows at slaughter (one month postpartum) by both traditional bacteriology and 16S rRNA gene sequencing. The bacteriology that we performed was specifically designed to identify living organisms in the reproductive tract. The strength of the bacteriology is that living organisms are identified. The weakness is that most microorganisms fail to grow when standard culture techniques are used [52]. The purpose of the 16S gene sequencing (metagenomic analysis) was to identify the entire microbiome that includes species that can be cultured as well as those that cannot. The weakness of 16S gene sequencing is that genera and species identification are based on the presence of nucleic acids and we cannot definitely say whether the identified organisms were intact and viable at the time of sample collection.

We sampled the outside of the reproductive tract as a positive control for bacterial contamination (likely arising from the environment during tissue collection). Using traditional bacteriology, we found that the original disease diagnosis and antibiotic treatment had no effect on the prevalence of the detected species for this external control sample (as expected). The bacterial species cultured on the external surface of the tract are typically found in the environment, skin, or gastrointestinal tract suggesting that they contaminated the tract when it was collected. Within the lumen of the uterine horn, however, we noted that bacteria that are typically found in the postpartum uterus (e.g. first week) were still present one month postpartum, particularly in cows with purulent flflush or an acute metritis-like infection (Tables 4 and 5). Approximately 50% of the cows that we originally diagnosed as healthy had “no growth” for the bacteriology. This percentage was less (17%) for the cows originally diagnosed as metritis. Most of the cows with purulent flflush (83%) and all of the cows with an acute infection had culturable bacteria in the uterine lumen. We cultured typical metritis-associated bacteria including *Fusobacterium necrophorum* (Fig. 5), *Trueperella pyogenes*, and *Helcococcus ovis*. Importantly, treating cows with antibiotic at disease diagnosis did not appear to systematically reduce the prevalence of cultured species within the individual cows (Table 4) with the caveat that this study would not detect small changes in the prevalence of bacterial species. Nonetheless, the conclusions from the bacterial culture (no effect of antibiotic treatment) were similar to the conclusions of the 16S rRNA gene sequencing (see below).

We sampled two different sites within the uterus (previously gravid and non-gravid horn) and we did not find any evidence that bacterial populations [either cultured (data not shown) or sequenced (Fig. 7A)] differed for these two locations. For subsequent analyses, therefore, we pooled data from the two sites. We did find a difference in the bacteria on the outside of the tract (external control) and inside of the tract (uterine lumen; Tables 3 and 4 and Fig. 7), as expected. Collectively, the results support the idea that the microbiome composition of the non-gravid and previously gravid uterine horns are similar, which is not surprising as both horns are connected through the uterine body which theoretically allows microbial migration from one horn to another. Furthermore, even though preventive measures were taken to maintain sterility of the outside of the reproductive tract following animal slaughter, we found that environmental contamination occurred during the process of harvesting the reproductive tract at the abattoir and transport to the laboratory for sample collection (conducted inside a clean biosafety cabinet).

Considering only samples collected from the uterine lumen, the number of 16S rRNA gene sequence reads at one month postpartum was greater for cows that had metritis at disease diagnosis compared with control and there was no effect of antibiotic treatment on the number of 16S rRNA gene reads (Fig. 8A). Furthermore, we found no discernible impact of antibiotic treatment on alpha or beta diversity metrics, and differential abundance analysis using ANCOM did not reveal any effect of antibiotic treatment. These results agree with the findings of the bacterial culture (Table 4) that the antibiotic treatment at disease diagnosis did not have a lasting effect on the uterine microbiome at one month postpartum.

We classified cows based on the contents of their uterine lumen (flflush phenotype) into either “clear” (clear flflushing fluid), “purulent” (pus in the flflushing fluid) or acutely infected (metritis-like appearance at one month postpartum). For this analysis, we noted a greater number of sequence reads for the cows with a purulent or acutely infected uterus (Fig. 9A). We also noted that the three distinct flflush phenotypes clustered separately on the Jaccard PCoA plot (Fig. 9B). We noted greater Pielou’s evenness in cows with an acute infection compared with a purulent lumen (Fig. 9C) demonstrating dysbiosis in cows with an acute infection relative to those with a purulent lumen. Cows with a clear uterine flflush had greater Faith’s Phylogenetic diversity (Fig. 9D) and greater unweighted Unifrac distances (Fig. 9E) compared with cows with purulent flflush and acute infection. These two measures indicate a more diverse microbiome (less similarity between the samples of individual cows) for cows with a clear uterine flush. Uterine disease (either endometritis or acute infection) at one month postpartum, therefore, was associated with less microbial diversity (dysbiosis due to select overgrowth of pathogenic bacteria).

In a final series of analyses, we performed multinomial regression of the uterine microbiome at one month postpartum using Songbird and TensorBoard. Because the initial microbial load of individual samples submitted for sequencing is unknown, the analytical approach implemented by Songbird [47] is based on ranking microbes with the greatest change relative to each other within a sample. Three distinct models were tested. The first model (“null model”) represented random chance and was compared with additional models containing selected variables presumed to be associated with uterine disease 30 days postpartum. The second model contained only the categorical information of flush phenotype and the third model (“full model”) was created containing variables representing the flush phenotype (clear, purulent, acute), cow identification, disease status one week postpartum (healthy versus metritis), antibiotic (treated versus not treated), and plasma progesterone concentrations at the time of slaughter (indicative of cyclicity). The prediction accuracy (capacity of the model to predict the observed read counts in samples) improved for the full model relative to the null and categorical models (Figs. 10A and 10B). There were a total of 78 features (ASV) ranked out of 1,560 features detected at one month postpartum. The ASV that defined the most differently abundant features according to Songbird were used to create a “heatmap” where unsupervised hierarchical clustering was used to group individual samples from the gravid and non-gravid horns based on their microbiome. Using this analysis, we found that acute metritis and endometritis were defined by an entirely different microbiome (Fig. 11). Acute metritis, for example, was defined by dominant genera and species such as *Bacteroides, Fusobacterium necrophorum, Trueperella, Prevotella, Helcococcus ovis, Porphyromonas levii*, and *Campylobacter*. These species are well-established metritis pathogens [1, 14, 15, 53] and many were also shown to be differentially expressed at disease diagnosis in the present study (Fig. 4). Cows with an acute infection at one month postpartum, therefore, had been unable to recover from the original disease. Conversely, cows with a purulent lumen were defined by *Mycoplasmopsis bovigenitalium, Streptobacillus, Histophilus, Ureaplasma diversum*, and *Caviibacter*; bacterial genera and species that are also known to populate the uterus but are not necessarily associated with metritis. Indeed, *Ureaplasma diversum* was greater in healthy cows at disease diagnosis (Fig. 4). Cows that presented with a clear uterine flush clustered in the center of the diagram and failed to show any dominant bacterial species. This finding agrees generally with that of Fig. 9 where cows with a clear flush phenotype had a more diverse microbiome.

## Conclusions

We found that cows diagnosed with metritis and healthy control cows differed in the prevalence of cultured bacteria and 16S rRNA DNA sequence reads (greater in metritis cows). The antibiotic treatment, however, had no effect on the prevalence of cultured bacteria or sequenced metagenome. Likewise, the antibiotic treatment had no effect on the prevalence of endometritis (1 month postpartum) in cows that had been originally diagnosed as healthy or metritic. Cows readily switched between disease groups from disease diagnosis (7 to 10 d postpartum; healthy versus metritis) to final diagnosis (clear or purulent) at one month postpartum. To account for the complex study design and confounding variables known to affect the uterine microbiome, a multinomial regression analysis was performed. This analysis revealed distinct microbial communities associated with a healthy uterus (clear flush) or diseased uterus that presented as purulent or acutely infected at one month postpartum. Given the results of this study and the importance of endometritis to fertility, understanding the mechanisms through which specific bacterial species persist in cows that develop endometritis (regardless of whether they are initially metritis or healthy) is the most important question to be answered in future studies.

## Figures and Tables

**Table 1:** Number of cows that were either healthy or with metritis and treated with antibiotic (Ab) or not treated (No trt) with antibiotic at 7 to 10 days postpartum that were later classified as having either a clear uterine lumen, a purulent uterine lumen (endometritis) or having an acute uterine infection that resembled early postpartum metritis at time of slaughter (30 days postpartum).

	Healthy (n = 17)			Metritis (n = 18)		
Flush	Ab (n = 9)	No trt (n = 8)	Total (%)	Ab (n = 9)	No trt (n = 9)	Total (%)
Clear	6	6	12 (71)	4	4	8 (44)
Purulent	3	2	5 (29)	4	3	7 (39)
Acute infection	0	0	0	1	2	3 (17)
TOTAL	9	8	17 (100)	9	9	18 (100)

**Table 2:** Least square mean and SE for fibrosis, epithelium inflammation, stromal inflammation, and eosinophil score derived from a histological evaluation of uterine cross sections. Data are presented for healthy versus metritis that were either treated with antibiotic (Ab) or not treated (No trt) with antibiotic (top panel) and clear versus purulent uterine flush that were either Ab treated or not treated (bottom panel) at disease diagnosis.

	Healthy (n = 17)			Metritis (n = 18)		
Item^1^	Ab (n = 9)	No trt (n = 8)	SE	Ab (n = 9)	No trt (n = 9)	SE
Fibrosis score	2.44	2.67	0.12	2.52	2.59	0.10
Epithelial inflammation score^y^	0.87	1.15	0.10	1.21	1.00	0.08
Stromal inflammation score^z^	2.58	2.79	0.04	2.73	2.66	0.034
Eosinophil score^x^	0.94	1.33	0.17	1.76	1.40	0.14
	Clear (n = 17)			Purulent (n = 18)		
Item	Ab (n = 10)	No trt (n = 10)	SE	Ab (n = 8)	No trt (n = 7)	SE
Fibrosis score**	2.30	2.48	0.10	2.66	2.78	0.12
Epithelial inflammation score***	0.88	0.80	0.08	1.21	1.35	0.10
Stromal inflammation score**^,y^	2.65	2.62	0.03	2.66	2.84	0.04
Eosinophil score***	1.01	0.83	0.14	1.68	1.90	0.17

1 Histological scoring of endometrial fibrosis (1 to 3; mild to severe) and endometrial inflammatory cell or eosinophil scored from 0.5 (< 10 cells/field) to 3 (> 50 cells/field)]. Asterix (*P < 0.05, **P < 0.01, ***P < 0.001) is for the probability of the main effect (healthy versus metritis or clear versus purulent). Letter is for the interaction of the main effect with treatment (^x^*P* < 0.05, ^y^*P*<0.01, ^z^*P*<0.001).

**Table 3:** Partial list of cultured bacterial species isolated from the external surface of the reproductive tract following slaughter at one month postpartum for cows that were either healthy or metritis and treated with antibiotic (Ab) or not treated (No trt) with antibiotic. The list include species isolated from at least 8 cows.

	Healthy (n = 17)			Metritis (n = 18)		
Species	Ab (n = 9)	No trt (n = 8)	Total (%)	Ab (n = 9)	No trt n = 9	Total (%)
*Aerococcus viridans*	5	3	8 (47)	4	5	9 (50)
*Bacillus altitudinis*	2	3	5 (29)	4	2	6 (33)
*Bacillus licheniformis*	5	4	9 (53)	3	3	6 (33)
*Bacillus pumilus*	8	3	11 (65)	4	5	9 (50)
*Corynebacterium renale*	1	1	2 (12)	2	4	6 (33)
*Corynebacterium xerosis*	8	5	13 (76)	6	6	12 (67)
*Dietzia maris*	2	2	4 (24)	2	2	4 (22)
*Escherichia coli*	3	4	7 (41)	5	3	8 (44)
*Micrococcus luteus*	4	0	4 (24)	3	4	7 (39)
*Staphylococcus epidermidis*	8	5	13 (76)	5	8	13 (72)
*Staphylococcus pasteuri*	8	2	10 (59)	3	4	7 (39)
*Staphylococcus warneri*	3	2	5 (29)	1	4	5 (28)
*Streptococcus pluranimalium*	7	1	8 (47)	2	3	5 (28)
*Trueperella pyogenes*	3	1	4 (24)	3	1	4 (22)

**Table 4:** Partial list of cultured bacterial species isolated from the lumen of the uterine horn following slaughter at one month postpartum for cows that were either healthy or metritis and treated with antibiotic (Ab) or not treated (No trt) with antibiotic at 7 to 10 days postpartum. The list include species isolated from at least 3 cows.

	Healthy (n = 17)			Metritis (n = 18)		
Species	Ab (n = 9)	No trt (n = 8)	Total (%)	Ab (n = 9)	No trt (n = 9)	Total (%)
*Corynebacterium renale*	0	0	0 (0)	1	2	3 (17)
*Cutibacterium acnes*	1	0	1 (6)	1	1	2 (11)
*Fusobacterium necrophorum*	0	0	0 (0)	3	4	7 (39)
*Helcococcus ovis*	0	0	0 (0)	2	1	3 (17)
*Histophilus somni*	1	0	1 (6)	1	7	8 (44)
*Paenibacillus cookii*	0	1	1 (6)	1	1	2 (11)
*Peptoniphilus indolicus*	0	0	0 (0)	0	4	4 (22)
*Porphyromonas levii*	0	0	0 (0)	2	3	5 (28)
*Prevotella heparinolytica*	0	0	0 (0)	1	2	3 (17)
*Staphylococcus epidermidis*	0	1	1 (6)	2	0	2 (11)
*Streptococcus pluranimalium*	0	0	0 (0)	2	2	4 (22)
*Trueperella pyogenes*	1	0	1 (6)	4	5	9 (50)
No growth	3	5	8 (47)	2	1	3 (17)

**Table 5:** Partial list of cultured bacterial species isolated from the lumen of the uterine horn at one month postpartum for cows that were found to have a clear (non-purulent) uterine lumen, a purulent (pus-filled) uterine lumen or an acute uterine infection at the time of slaughter. The list include species isolated from at least 3 cows.

Species	Clear (%) n = 20	Punilent (%) n = 12	Acute (%) n = 3
*Corynebacterium renale*	2 (10)	1 (8)	0 (0)
*Cutibacterium acnes*	2 (10)	1 (8)	0 (0)
*Fusobacterium necrophorum*	0 (0)	4 (33)	3 (100)
*Helcococcus ovis*	0 (0)	2 (17)	1 (33)
*Histophilus somni*	3 (15)	4 (33)	2 (67)
*Paenibacillus cookii*	1 (5)	2 (17)	0 (0)
*Peptoniphilus indolicus*	0 (0)	2 (17)	2 (67)
*Porphyromonas levii*	0 (0)	3 (25)	2 (67)
*Prevotella heparinolytica*	0 (0)	2 (17)	1 (33)
*Staphylococcus epidermidis*	3 (15)	0 (0)	0 (0)
*Streptococcus pluranimalium*	1 (5)	1 (8)	2 (67)
*Trueperella pyogenes*	2 (10)	5 (42)	3 (100)
No growth	9 (45)	2 (17)	0 (0)

## Data Availability

The sequence files and associated metadata for all samples utilized in this study have been securely deposited in the NCBI Sequence Read Archive (SRA) repository (BioProject accession: PRJNA1096992; Reviewer link: https://dataview.ncbi.nlm.nih.gov/object/PRJNA1096992? reviewer=bmqip7hhph2punna5hqs9ofmj). Original QIIME2, Songbird, Qurro, and BLAST scripts are available on GitHub (https://github.com/pyjm/Moraes_Lucy_postartum_microbiome).

## References

[1] Bromfield JJ, Santos JEP, Block J, Williams RS, Sheldon IM. PHYSIOLOGY AND ENDOCRINOLOGY SYMPOSIUM: Uterine infection: linking infection and innate immunity with infertility in the high-producing dairy cow. J Anim Sci 2015; 93:2021–2033. 26020298 10.2527/jas.2014-8496

[2] LeBlanc SJ. Review: Relationships between metabolism and neutrophil function in dairy cows in the peripartum period. Animal 2020; 14:s44–s54. 32024567 10.1017/S1751731119003227

[3] Sheldon IM, Molinari PCC, Ormsby TJR, Bromfield JJ. Preventing postpartum uterine disease in dairy cattle depends on avoiding, tolerating and resisting pathogenic bacteria. Theriogenology 2020; 150:158–165. 31973964 10.1016/j.theriogenology.2020.01.017 PMC7234917

[4] Rajala PJ, Gröhn YT. Effects of Dystocia, Retained Placenta, and Metritis on Milk Yield in Dairy Cows. Journal of Dairy Science 1998; 81:3172–3181. 9891264 10.3168/jds.s0022-0302(98)75883-7

[5] Garzon A, Habing G, Lima F, Silva-Del-Rio N, Samah F, Pereira R. Defining clinical diagnosis and treatment of puerperal metritis in dairy cows: A scoping review. J Dairy Sci 2022; 105:3440–3452. 35151476 10.3168/jds.2021-21203

[6] Pérez-Báez J, Silva TV, Risco CA, Chebel RC, Cunha F, De Vries A, Santos JEP, Lima FS, Pinedo P, Schuenemann GM, Bicalho RC, Gilbert RO, The economic cost of metritis in dairy herds. J Dairy Sci 2021; 104:3158–3168. 33455790 10.3168/jds.2020-19125

[7] Wagener K, Gabler C, Drillich M. A review of the ongoing discussion about definition, diagnosis and pathomechanism of subclinical endometritis in dairy cows. Theriogenology 2017; 94:21–30. 28407857 10.1016/j.theriogenology.2017.02.005

[8] Hagner KA, Nordgren HS, Aaltonen K, Sarjokari K, Rautala H, Sironen T, Sukura A, Rajala-Schultz PJ. Necropsy-based study on dairy cow mortality—Underlying causes of death. Journal of Dairy Science 2023; 106:2846–2856. 36870842 10.3168/jds.2022-22466

[9] LeBlanc SJ, Duffield TF, Leslie KE, Bateman KG, Keefe GP, Walton JS, Johnson WH. Defining and Diagnosing Postpartum Clinical Endometritis and its Impact on Reproductive Performance in Dairy Cows. Journal of Dairy Science 2002; 85:2223–2236. 12362455 10.3168/jds.S0022-0302(02)74302-6

[10] Koyama T, Omori R, Koyama K, Matsui Y, Sugimoto M. Optimization of diagnostic methods and criteria of endometritis for various postpartum days to evaluate infertility in dairy cows. Theriogenology 2018; 119:225–232. 30055393 10.1016/j.theriogenology.2018.07.002

[11] Bruinjé TC, Morrison EI, Ribeiro ES, Renaud DL, LeBlanc SJ. Associations of inflammatory and reproductive tract disorders postpartum with pregnancy and early pregnancy loss in dairy cows. Journal of Dairy Science 2024; 107:1630–1644. 37820756 10.3168/jds.2023-23976

[12] LeBlanc SJ. Review: Postpartum reproductive disease and fertility in dairy cows. Animal 2023; 17:100781. 37567665 10.1016/j.animal.2023.100781

[13] Moraes JGN, Silva PRB, Mendonça LGD, Okada CTC, Chebel RC. Risk factors for purulent vaginal discharge and its association with reproductive performance of lactating Jersey cows. J Dairy Sci 2021; 104:12816–12829. 34482979 10.3168/jds.2021-20502

[14] Jeon SJ, Vieira-Neto A, Gobikrushanth M, Daetz R, Mingoti RD, Parize ACB, de Freitas SL, da Costa ANL, Bicalho RC, Lima S, Jeong KC, Galvão KN. Uterine Microbiota Progression from Calving until Establishment of Metritis in Dairy Cows. Applied and Environmental Microbiology 2015; 81:6324–6332. 26150453 10.1128/AEM.01753-15 PMC4542247

[15] Carneiro LC, Cronin JG, Sheldon IM. Mechanisms linking bacterial infections of the bovine endometrium to disease and infertility. Reprod Biol 2016; 16:1–7. 26952747 10.1016/j.repbio.2015.12.002

[16] Haimerl P, Heuwieser W. Invited review: Antibiotic treatment of metritis in dairy cows: a systematic approach. J Dairy Sci 2014; 97:6649–6661. 25218751 10.3168/jds.2014-8462

[17] Chenault JR, McAllister JF, Chester ST, Dame KJ, Kausche FM, Robb EJ. Efficacy of ceftiofur hydrochloride sterile suspension administered parenterally for the treatment of acute postpartum metritis in dairy cows. Journal of the American Veterinary Medical Association 2004; 224:1634–1639. 15154734 10.2460/javma.2004.224.1634

[18] Jeon SJ, Cunha F, Daetz R, Bicalho RC, Lima S, Galvão KN. Ceftiofur reduced Fusobacterium leading to uterine microbiota alteration in dairy cows with metritis. Animal Microbiome 2021; 3:15. 33509303 10.1186/s42523-021-00077-5 PMC7844903

[19] McLaughlin CL, Stanisiewski E, Lucas MJ, Cornell CP, Watkins J, Bryson L, Tena JKS, Hallberg J, Chenault JR. Evaluation of two doses of ceftiofur crystalline free acid sterile suspension for treatment of metritis in lactating dairy cows. J Dairy Sci 2012; 95:4363–4371. 22818449 10.3168/jds.2011-5111

[20] Haimerl P, Arlt S, Borchardt S, Heuwieser W. Antibiotic treatment of metritis in dairy cows-A meta-analysis. J Dairy Sci 2017; 100:3783–3795. 28365115 10.3168/jds.2016-11834

[21] Merenda VR, Lezier D, Odetti A, Figueiredo CC, Risco CA, Bisinotto RS, Chebel RC. Effects of metritis treatment strategies on health, behavior, reproductive, and productive responses of Holstein cows. J Dairy Sci 2021; 104:2056–2073. 33309374 10.3168/jds.2020-19076

[22] Lima FS, Vieira-Neto A, Snodgrass JA, De Vries A, Santos JEP. Economic comparison of systemic antimicrobial therapies for metritis in dairy cows. Journal of Dairy Science 2019; 102:7345–7358. 31178192 10.3168/jds.2018-15383

[23] Silva TV, de Oliveira EB, Pérez-Báez J, Risco CA, Chebel RC, Cunha F, Daetz R, Santos JEP, Lima FS, Jeong KC, Galvão KN. Economic comparison between ceftiofur-treated and nontreated dairy cows with metritis. Journal of Dairy Science 2021; 104:8918–8930. 33934874 10.3168/jds.2020-19430

[24] Kelton DF, Lissemore KD, Martin RE. Recommendations for recording and calculating the incidence of selected clinical diseases of dairy cattle. J Dairy Sci 1998; 81:2502–2509. 9785242 10.3168/jds.S0022-0302(98)70142-0

[25] Pohler KG, Geary TW, Johnson CL, Atkins JA, Jinks EM, Busch DC, Green JA, MacNeil MD, Smith MF. Circulating bovine pregnancy associated glycoproteins are associated with late embryonic/fetal survival but not ovulatory follicle size in suckled beef cows. J Anim Sci 2013; 91:4158–4167. 23825331 10.2527/jas.2013-6348

[26] Yu Z, Morrison M. Improved extraction of PCR-quality community DNA from digesta and fecal samples. Biotechniques 2004; 36:808–812. 15152600 10.2144/04365ST04

[27] Caporaso JG, Lauber CL, Walters WA, Berg-Lyons D, Lozupone CA, Turnbaugh PJ, Fierer N, Knight R. Global patterns of 16S rRNA diversity at a depth of millions of sequences per sample. Proc Natl Acad Sci U S A 2011; 108 Suppl 1:4516–4522. 20534432 10.1073/pnas.1000080107 PMC3063599

[28] Bolyen E, Rideout JR, Dillon MR, Bokulich NA, Abnet CC, Al-Ghalith GA, Alexander H, Alm EJ, Arumugam M, Asnicar F, Bai Y, Bisanz JE, Reproducible, interactive, scalable and extensible microbiome data science using QIIME 2. Nat Biotechnol 2019; 37:852–857. 31341288 10.1038/s41587-019-0209-9 PMC7015180

[29] Martin M. Cutadapt removes adapter sequences from high-throughput sequencing reads. EMBnet j 2011; 17:10.

[30] Callahan BJ, McMurdie PJ, Rosen MJ, Han AW, Johnson AJA, Holmes SP. DADA2: High-resolution sample inference from Illumina amplicon data. Nat Methods 2016; 13:581–583. 27214047 10.1038/nmeth.3869 PMC4927377

[31] Pielou EC. The measurement of diversity in different types of biological collections. Journal of Theoretical Biology 1966; 13:131–144.

[32] Faith DP. Conservation evaluation and phylogenetic diversity. Biological Conservation 1992; 61:1–10.

[33] Lozupone C, Knight R. UniFrac: a New Phylogenetic Method for Comparing Microbial Communities. AEM 2005; 71:8228–8235. 10.1128/AEM.71.12.8228-8235.2005 PMC1317376 16332807

[34] Lozupone CA, Hamady M, Kelley ST, Knight R. Quantitative and qualitative beta diversity measures lead to different insights into factors that structure microbial communities. Appl Environ Microbiol 2007; 73:1576–1585. 17220268 10.1128/AEM.01996-06 PMC1828774

[35] Jaccard P. The distribution of the flora in the alpine zone. 1. New Phytologist 1912; 11:37–50.

[36] Bray JR, Curtis JT. An ordination of the upland forest communities of southern Wisconsin. Ecological Monographs 1957; 27:326–349.

[37] Vázquez-Baeza Y, Pirrung M, Gonzalez A, Knight R. EMPeror: a tool for visualizing high-throughput microbial community data. GigaSci 2013; 2:16. 10.1186/2047-217X-2-16 PMC4076506 24280061

[38] Vázquez-Baeza Y, Gonzalez A, Smarr L, McDonald D, Morton JT, Navas-Molina JA, Knight R. Bringing the Dynamic Microbiome to Life with Animations. Cell Host & Microbe 2017; 21:7–10. 28081445 10.1016/j.chom.2016.12.009

[39] Bokulich N, Robeson M, Kaehler B, Dillon M. bokulich-lab/RESCRIPt: 2020.6.1 2020.

[40] Pruesse E, Quast C, Knittel K, Fuchs BM, Ludwig W, Peplies J, Glockner FO. SILVA: a comprehensive online resource for quality checked and aligned ribosomal RNA sequence data compatible with ARB. Nucleic Acids Res 2007; 35:7188–7196. 17947321 10.1093/nar/gkm864 PMC2175337

[41] Pedregosa F, Varoquaux G, Gramfort A, Michel V, Thirion B, Grisel O, Blondel M, Prettenhofer P, Weiss R, Dubourg V, Vanderplas J, Passos A, Scikit-learn: Machine Learning in Python. Journal of Machine Learning Research 2011; 12:2825–2830.

[42] Quast C, Pruesse E, Yilmaz P, Gerken J, Schweer T, Yarza P, Peplies J, Glockner FO. The SILVA ribosomal RNA gene database project: improved data processing and web-based tools. Nucleic Acids Res 2013; 41:D590–6. 23193283 10.1093/nar/gks1219 PMC3531112

[43] Bokulich NA, Kaehler BD, Rideout JR, Dillon M, Bolyen E, Knight R, Huttley GA, Gregory Caporaso J. Optimizing taxonomic classification of marker-gene amplicon sequences with QIIME 2’s q2-feature-classifier plugin. Microbiome 2018; 6:90. 29773078 10.1186/s40168-018-0470-z PMC5956843

[44] McDonald D, Clemente JC, Kuczynski J, Rideout JR, Stombaugh J, Wendel D, Wilke A, Huse S, Hufnagle J, Meyer F, Knight R, Caporaso JG. The Biological Observation Matrix (BIOM) format or: how I learned to stop worrying and love the ome-ome. GigaSci 2012; 1:7. 10.1186/2047-217X-1-7 PMC3626512 23587224

[45] Mandal S, Van Treuren W, White RA, Eggesbø M, Knight R, Peddada SD. Analysis of composition of microbiomes: a novel method for studying microbial composition. Microbial Ecology in Health & Disease 2015; 26. 10.3402/mehd.v26.27663 PMC4450248 26028277

[46] Cantrell K, Fedarko MW, Rahman G, McDonald D, Yang Y, Zaw T, Gonzalez A, Janssen S, Estaki M, Haiminen N, Beck KL, Zhu Q, EMPress Enables Tree-Guided, Interactive, and Exploratory Analyses of Multi-omic Data Sets. mSystems 2021; 6:e01216–20. 33727399 10.1128/mSystems.01216-20 PMC8546999

[47] Morton JT, Marotz C, Washburne A, Silverman J, Zaramela LS, Edlund A, Zengler K, Knight R. Establishing microbial composition measurement standards with reference frames. Nat Commun 2019; 10:2719. 31222023 10.1038/s41467-019-10656-5 PMC6586903

[48] Abadi M, Barham P, Chen J, Chen Z, Davis A, Dean J, Devin M, Ghemawat S, Irving G, Isard M, Kudlur M, Levenberg J, TensorFlow: A system for large-scale machine learning n.d.

[49] Fedarko MW, Martino C, Morton JT, González A, Rahman G, Marotz CA, Minich JJ, Allen EE, Knight R. Visualizing ‘omic feature rankings and log-ratios using Qurro. NAR Genomics and Bioinformatics 2020; 2. 10.1093/nargab/lqaa023 PMC7194218 32391521

[50] Garcia MM, Becker SA, Brooks BW, Berg JN, Finegold SM. Ultrastructure and molecular characterization of Fusobacterium necrophorum biovars. Can J Vet Res 1992; 56:318–325. 1477801 PMC1263563

[51] Huzzey JM, Duffield TF, LeBlanc SJ, Veira DM, Weary DM, von Keyserlingk MAG. Short communication: Haptoglobin as an early indicator of metritis. Journal of Dairy Science 2009; 92:621–625. 19164673 10.3168/jds.2008-1526

[52] Hilton SK, Castro-Nallar E, Pérez-Losada M, Toma I, McCaffrey TA, Hoffman EP, Siegel MO, Simon GL, Johnson WE, Crandall KA. Metataxonomic and Metagenomic Approaches vs. Culture-Based Techniques for Clinical Pathology. Front Microbiol 2016; 7:484. 27092134 10.3389/fmicb.2016.00484 PMC4823605

[53] Jeon SJ, Galvão KN. An Advanced Understanding of Uterine Microbial Ecology Associated with Metritis in Dairy Cows. Genomics Inform 2018; 16. 10.5808/GI.2018.16.4.e21 PMC6440669 30602082

